# Enhancing IgG fragment crystallizable sialylation improves the therapeutic activity of IL-23 cytokine blockade

**DOI:** 10.1172/jci.insight.198630

**Published:** 2026-02-19

**Authors:** Sebastian Kämpf, Marjan Hematianlarki, Leon Altmann, Jessica M. Bright, Alyssa M.A. Toda, Zohreh Mirzapoor, Valentin Zollner, Anja Werner, Johanna Bulang, Barbara Radovani, Miriam Wöhner, William Avery, Mark J. Karbarz, Pamela B. Conley, Greg P. Coffey, Falk Nimmerjahn

**Affiliations:** 1 Division of Genetics, Department of Biology, Friedrich Alexander University Erlangen-Nürnberg, Germany.; 2 Nuvig Therapeutics Inc., Palo Alto, California, USA.

**Keywords:** Autoimmunity, Immunology, Inflammation, Arthritis, Autoimmune diseases, Cytokines

## Abstract

The manuscript demonstrates that the activity of cytokine blocking antibodies can be enhanced by introducing a highly sialylated IgG Fc-domain.

**To the editor:** Cytokines are major drivers of chronic autoimmune inflammatory diseases including rheumatoid arthritis (RA), psoriasis, and different forms of chronic inflammatory bowel disease (IBD) or neuroinflammatory diseases (1). Consistent with this central role of specific signature cytokines in autoimmune inflammation, the use of monoclonal antibodies blocking the binding of proinflammatory cytokines to their respective receptors or the use of soluble decoy cytokine receptors has shown impressive clinical results. However, not all patients may have the same underlying cytokine response at a given state of their disease, which may explain at least in part why some patients do not respond to cytokine blockade (1). Thus, combining the activity of cytokine neutralization with other immunomodulatory pathways may help to enhance the therapeutic activity of cytokine blockade.

In this study, we have focused on IL-23–dependent inflammation and incorporated the F241A mutation in the Fc-domain of an IL-23 (p19) specific human IgG1 antibody, which was further hypersialylated with α2,6 linked sialic acid residues (Figure 1A and Supplemental Figure 1, A and B; and Supplemental Methods; supplemental material available online with this article; https://doi.org/10.1172/jci.insight.198630DS1) (2). As shown in Supplemental Figure 1C, introduction of the highly sialylated F241A Fc domain into the IL-23–specific human IgG1 antibody did not affect the binding to IL-23 and had a minimal effect on pharmacokinetics (PK) (Supplemental Figure 1, D and E). Since our previous studies have demonstrated that IL-23 blockade as well as IVIg therapy were efficacious in spontaneous or induced models of autoantibody-mediated rheumatoid arthritis (RA) and T cell–mediated experimental autoimmune encephalomyelitis (EAE), we focused on these preclinical model systems (3, 4). To demonstrate that the highly sialylated F241A Fc domain has an additive effect on IL-23 blockade, we used the IL-23–specific antibody at doses with no (50 mg/kg) or minimal therapeutic activity (150 mg/kg) (Figures 1, B–E, and Supplemental Figure 2). Indeed, the highly sialylated F241A containing IL-23–blocking antibody showed superior activity and suppressed arthritis symptoms and also blocked bone erosions and inflammatory osteoclastogenesis down to a dose of 25 mg/kg (Figure 1, B–E, and Supplemental Figure 2). Of note, the 10 mg/kg dose of the IL-23–IgG1–F241A antibody substantially reduced inflammatory osteoclastogenesis and, accordingly, the severity of bone erosions (Figures 1, D and E, and Supplemental Figure 2).

To study if the combination of both antiinflammatory mechanisms within 1 molecule is advantageous over coadministration of 2 separate therapeutics (IL-23–blocking antibody plus the sialylated F241A containing IgG-Fc), we treated mice with either 50 mg/kg of IL-23 IgG1 antibody, 50 mg/kg IgG1-F241A-Fc, the combination of both, or the IL-23–F241A antibody (Figure 1F). Since the sialylated F241A containing IgG-Fc has a lower half-life compared with the intact IgG antibodies, we used a molar excess to compensate for the reduced half-life (Supplemental Figure 1E). Whereas the IL-23–specific IgG1 antibody had no therapeutic activity at this dose, the treatment with the IgG1-F241A-Fc showed a transient amelioration of disease activity (Figures 1F and Supplemental Figure 3). In comparison, the combination of the anti–IL-23–IgG1 antibody with the IgG1-F241A-Fc resulted in a slightly enhanced and longer-lasting antiinflammatory effect. However, none of these treatment schemes reached the therapeutic efficacy of the IL-23–IgG1-F241A antibody, suggesting that incorporation of both mechanisms within 1 molecule is beneficial for an optimal therapeutic activity (Figures 1F and Supplemental Figure 3).

To broaden the relevance of our findings, we tested the therapeutic activity of the IL-23–IgG1–F241A antibody in an EAE model system. Animals were treated after the appearance of the first clinical symptoms of EAE with 25 mg/kg of the IL-23–IgG1 antibody, the TNP-IgG1-F241A, and the IL-23–IgG1-F241A antibody. As shown in Supplemental Figure 4, A and B, the IL-23–IgG1–F241A antibody was superior to the IL-23–IgG1 and the TNP-IgG1-F241A antibody to ameliorate disease symptoms. Moreover, doses of 10 as well as 5 mg/kg of the anti–IL-23–IgG1–F241A antibody were still able to suppress EAE symptoms. However, at the level of preventing nerve demyelination, all antibodies including the sialylated TNP-specific F241A-IgG1 antibody demonstrated protective effects (Supplemental Figure 4, C and D), consistent with the notion that, in the EAE model system, highly sialylated IgG antibodies as well as IL-23 blockade have strong therapeutic effects (2, 3, 5).

Collectively, our data support the notion that the combination of cytokine blockade with IgG Fc immunomodulatory mechanisms provides a promising therapeutic avenue to improve the therapy of patients with chronic autoimmune inflammatory diseases.

## Funding support

This study was funded by NUVIG therapeutics and grants from the German Research Foundation (DFG-FOR2886-B2, DFG-FOR2953-P3, DFG-TRR369-C01) to FN.

## Supplementary Material

Supplemental data

## Figures and Tables

**Figure 1:**
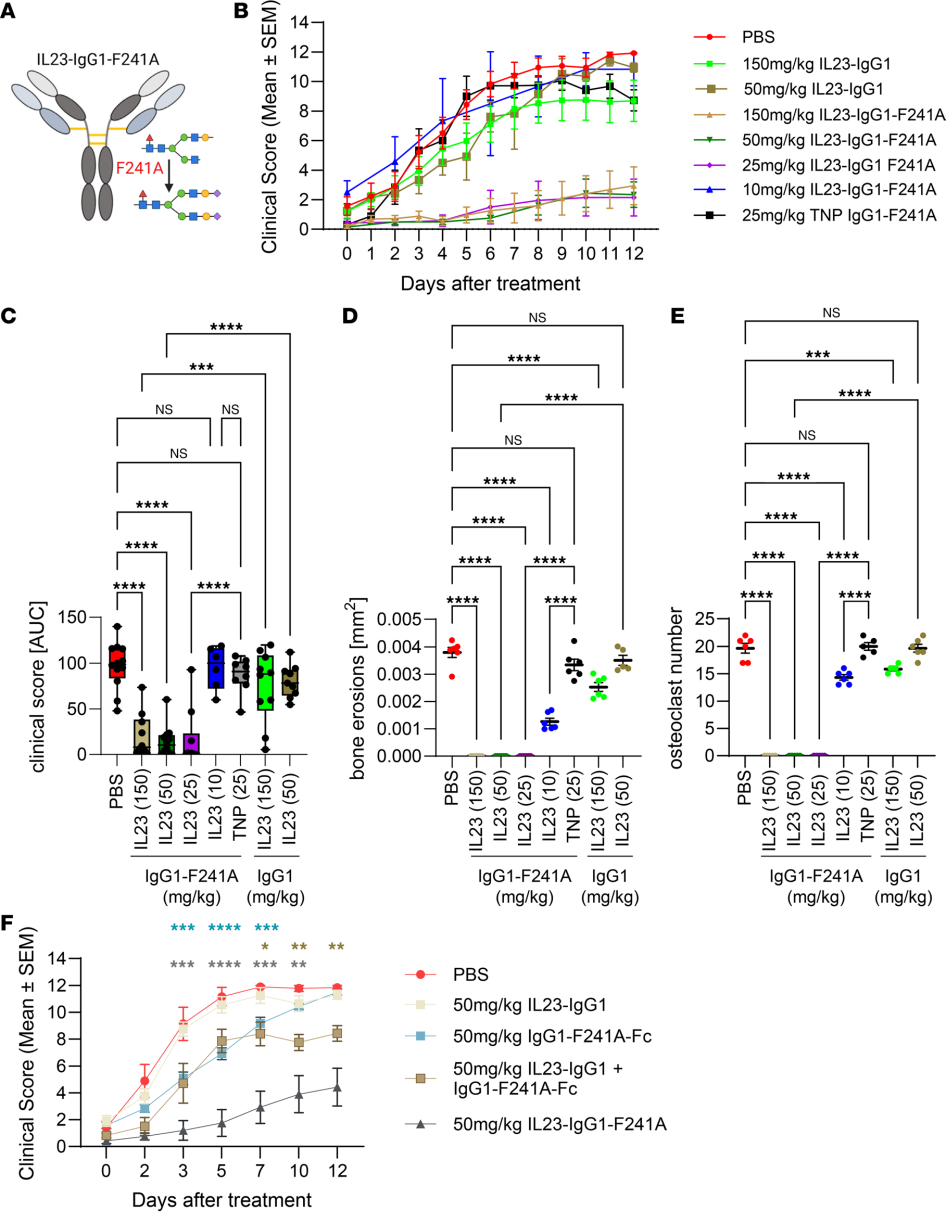
Immunomodulatory activity of IL-23–specific antibody variants in rheumatoid arthritis. (**A**) Schematic representation of the IL-23–specific human hypersialylated (α2,6) IgG1 antibody with the F241A mutation (IL-23–IgG1–F241A). (**B** and **C**) Clinical scores (mean ± SEM) (**B**) or area under the curve (AUC) of clinical scores (box-and-whiskers plot) (**C**) of joint inflammation in KBxN mice treated with PBS or the indicated doses of human IL-23–IgG1, IL-23–IgG1–F241A, or TNP-specific IgG1-F241A antibodies. Depicted is the combination of 2 independent experiments with *n* = 6–12 mice per group. For statistical analysis, an ordinary 1-way ANOVA and a Dunnett’s multiple-comparison test were used. ***P < 0.001, ****P < 0.0001. (**D** and **E**) Quantification of bone erosions (in mm^2^) (**D**) and of the number of TRAP^+^ osteoclasts (**E**) on the surface of bone erosions in histological samples of KBxN mice treated with PBS or the respective amounts of the IL-23–IgG1, IL-23–IgG1–F241A, or TNP-IgG1-F241A antibody. Shown are all data points and the mean ± SEM of the combination of 2 independent experiments (*n* = 5–6 mice per group). For statistical analysis, a 2-way ANOVA and a Tukey’s multiple comparisons test were used. ***P < 0.001, ****P < 0.0001. (**F**) Clinical scores of KBxN mice treated with the indicated amounts of IL-23–IgG1, IgG1-F241A-Fc, IL-23–IgG1 + IgG1-F241A-Fc, IL-23–IgG1–F241A, or PBS. Statistically significant differences against PBS treatment were identified by using a 2-way ANOVA and Šídák’s multiple-comparison test. Depicted is the mean ± SEM of the combination of 2 independent experiments (*n* = 5–10 mice per group). *P < 0.05, **P < 0.01, ***P < 0.001, ****P < 0.0001.

## Version 1 02/19/2026

In-Press Preview

## Version 2 04/08/2026

Electronic publication
