# Supplementary material for: Enhancing IgG fragment crystallizable sialylation improves the therapeutic activity of IL-23 cytokine blockade
Source: JCI Insight. 2026 Feb 19;11(7):e198630. doi: 10.1172/jci.insight.198630 (PMC13134717; doi:10.1172/jci.insight.198630)
Supplement: Supplemental data [file jciinsight-11-198630-s100.pdf]

Kämpf et al., Supplemental Figures and Methods.

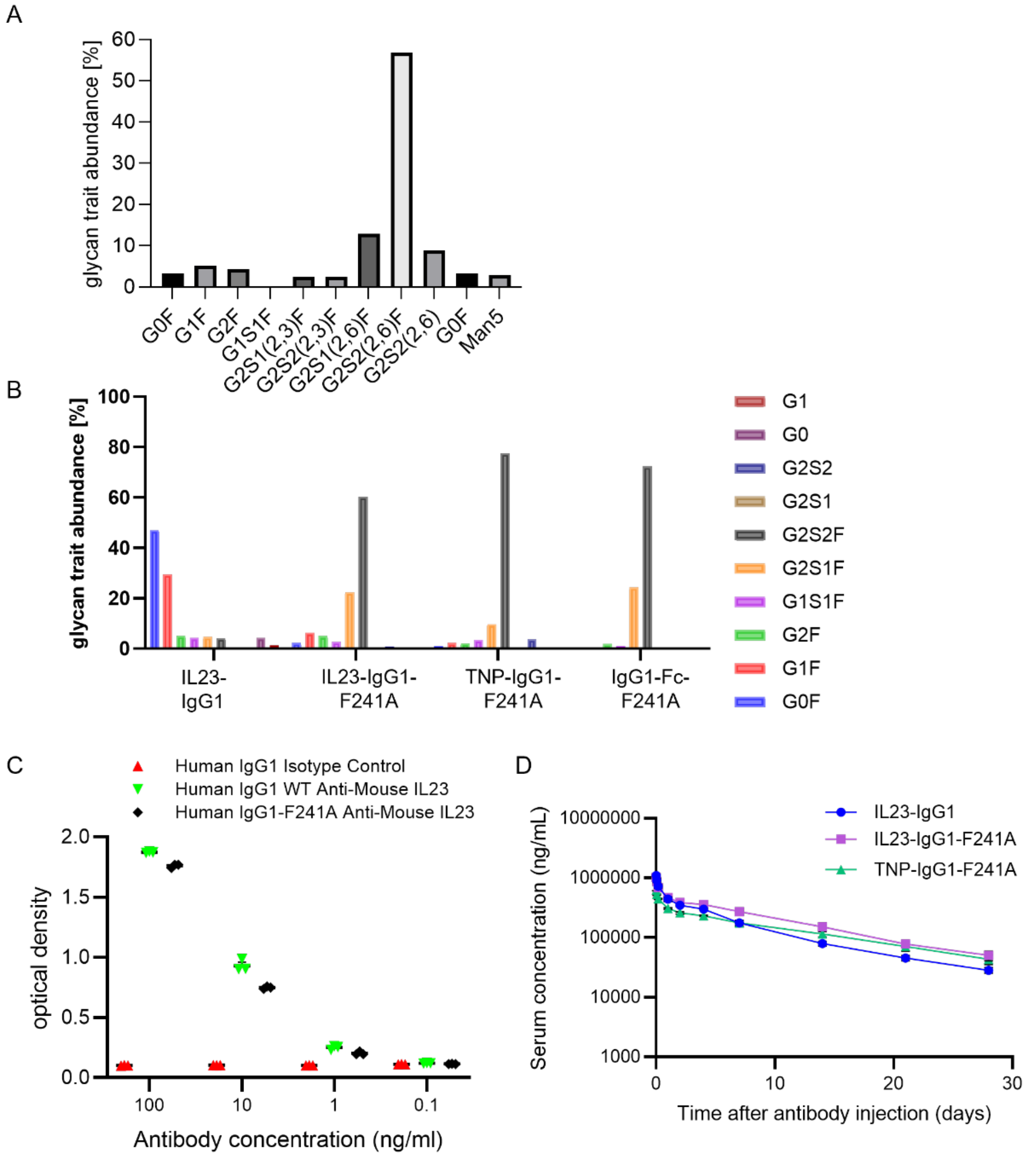

E

Group	T1/2 (d)	Cmax (ng/mL)	SE_Cmax	AUClast	SE_AUClast
IL23-IgG1-F241A	8.35	1 017 461,30	27 738,55	5 447 403,31	201 409,98
IL23-IgG1	9.35	1 094 633,77	45 545,16	3 968 743,00	99 992,69
TNP-IgG1-F241A	10.00	625 303,22	14 533,25	3 842 613,20	168 000,53
IgG1-Fc-F241A	4.1	1 043 622.38	83 555,31	1 063 837.66	143 727.05

Figure S1: Characterization of antibody variants used in vivo.

(**A**) Shown is the relative abundance in percent of the indicated glycan traits in the IL23-IgG1-F241A antibody preparation as determined by hydrophilic interaction liquid

chromatography (HILIC) high performance liquid chromatography (HPLC). G: galactose, F: fucose, S: Sialic acid, Man: mannose, 2-3: α2-3 linked sugar residue, 2-6: α2-6 linked sugar residue.

(**B**) Shown is the relative abundance in percent of the indicated glycan traits in the IL23-IgG1, IL23-IgG1-F241A, TNP-IgG1-F241A, or IgG1-Fc-F241A antibody preparations as determined by nanoLC-ESI-MS analysis. G: galactose, F: fucose, S: sialic acid.

(**C**) Shown is the binding (individual data points with mean+/-SEM)) of the indicated concentrations (n=3) of IL23-IgG1, IL23-IgG1-F241A, and a TNP-specific isotype control antibody (TNP-IgG1-F241A) to IL23 as determined by ELISA.

(**D**) Depicted is the serum concentration (ng/ml) of the indicated antibodies or antibody fragments at different time-points after injection. Shown is the mean +/- SEM of n=6 mice per group.

(**E**) The table depicts the half-life in days (T1/2 (d)), the maximal serum concentration (Cmax (ng/ml)), the standard error (SE) of Cmax, the area under the curve of the respective serum concentrations over time (AUClast) and the SE of the AUClast of the indicated human antibodies or Fc-fragments.

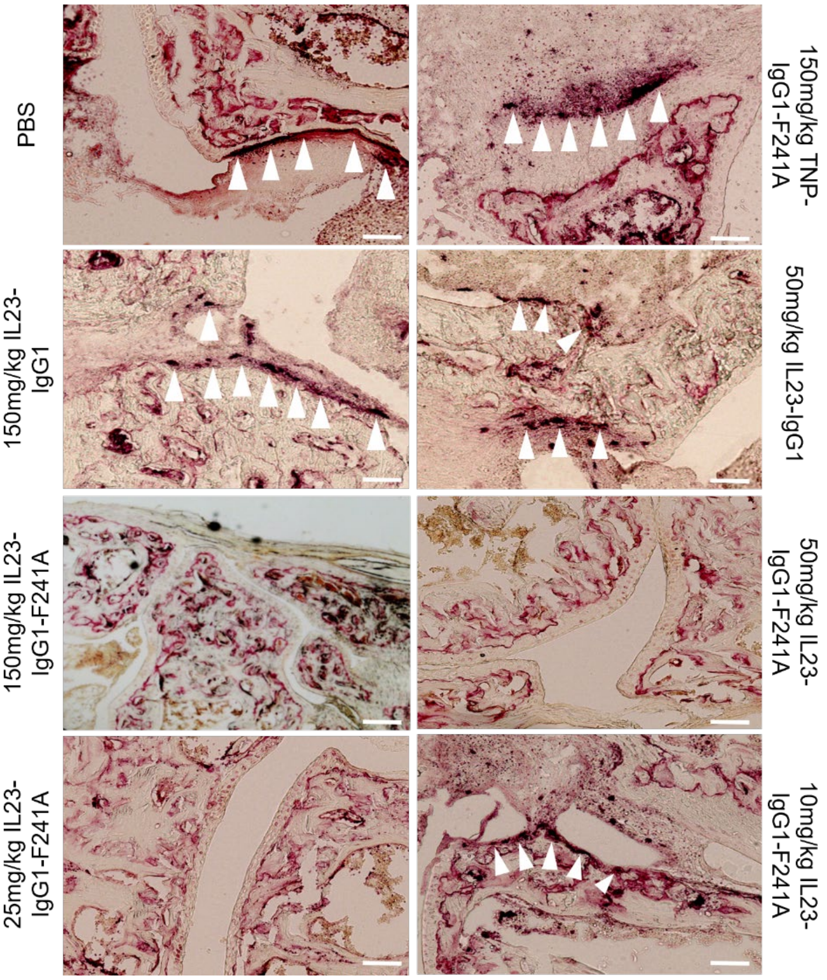

Figure S2: Impact of the IL23-F241A-Fc domain on bone pathology in KBxN mice.

Depicted are representative bone tissue sections of mice treated with PBS or with the indicated doses of TNP-IgG1-F241A, IL23-IgG1, or IL23-IgG1-F241A antibodies at day 12 after treatment initiation. Sections are stained for tartrate resistant acid phosphatase to detect osteoclasts. Arrows indicate TRAP+ osteoclasts. Scale bar represents 100μM.

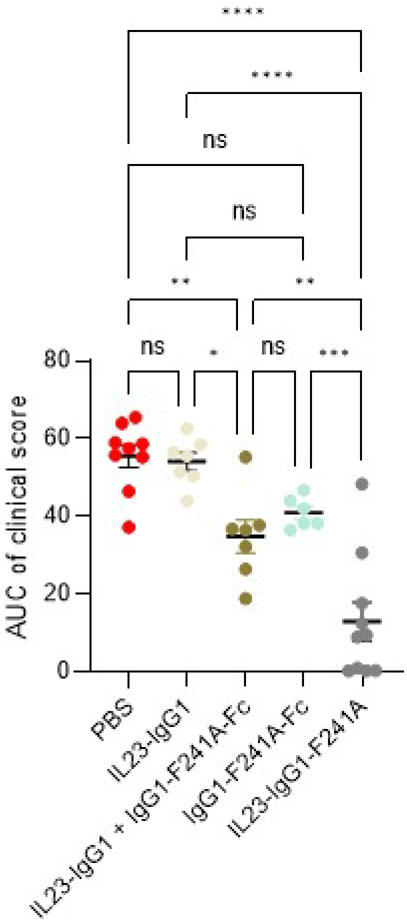

Figure S3: Impact of IL23 antibody intrinsic versus extrinsic F241A Fc-domains on rheumatoid arthritis activity.

Shown is the area under the curve (AUC) of the clinical score of KBxN mice treated with PBS or 50mg/kg of IL23-IgG1, IL23-IgG1 + IgG1-F241A-Fc, IgG1-F241A-Fc or IL23-IgG1-F241A (n=5-10 mice per group). Statistically significant differences were identified by using an ordinary one-way ANOVA and a Tukey's multiple comparison test. *p<0.05, **p<0.01, ***p<0.001, ****p<0.0001, n.s. not significant.

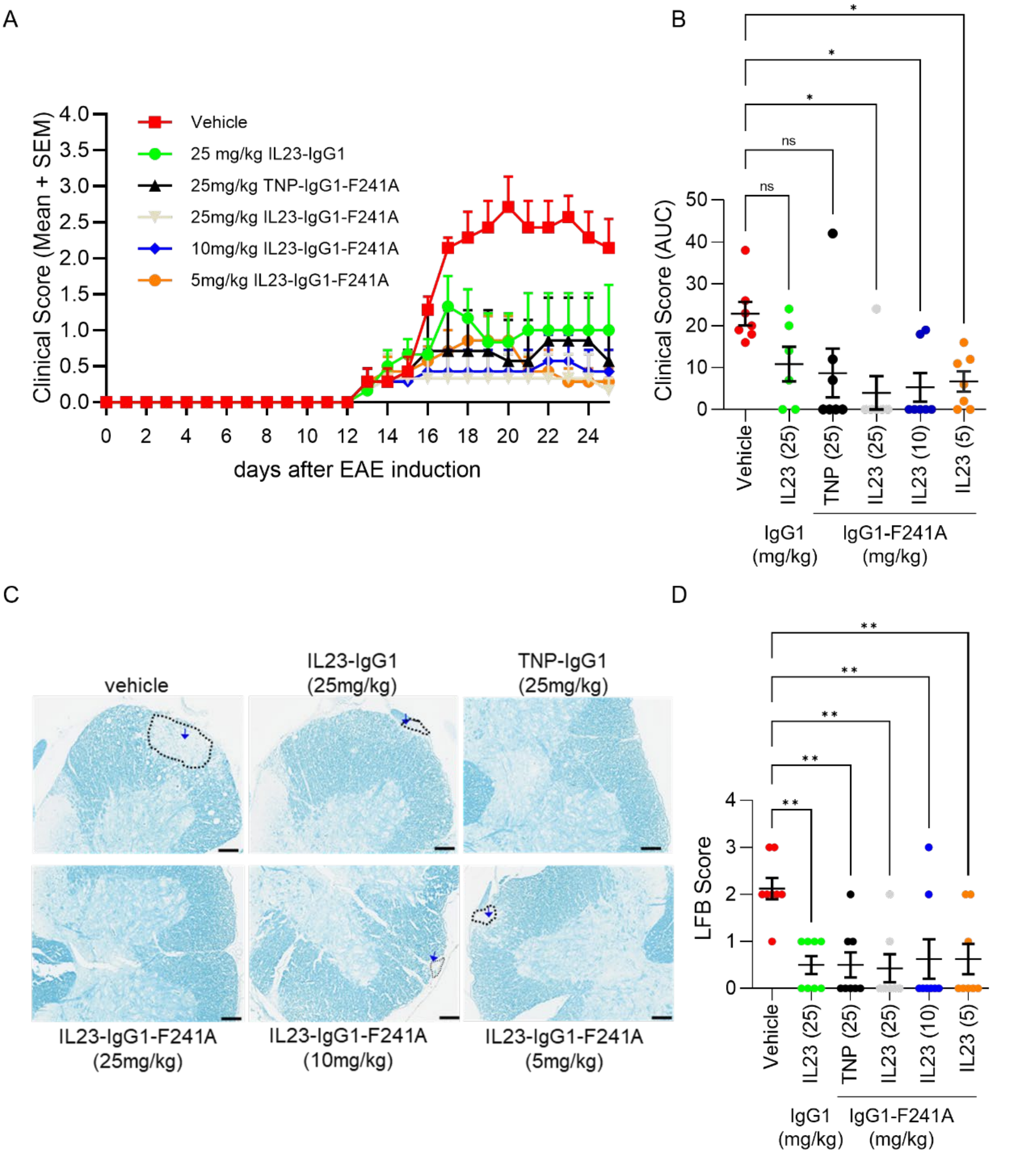

Figure S4: Immunomodulatory activity of IL23-IgG1 variants in experimental autoimmune encephalitis.

(**A**) Shown is the mean clinical score (mean +SEM) of mice (n=6-7) at the indicated time-points after EAE induction (day 0) followed by treatment with vehicle or the indicated amounts of IL23-IgG1, IL23-IgG1-F241A, or TNP-IgG1-F241A antibody starting at day 13 after induction of disease.

(**B**) Depicted is the area under the curve (AUC) of the clinical score (mean+SEM) of mice (n=6-7) treated with the indicated doses of the indicated antibodies or vehicle as a control. Statistical

significance was assessed by a mixed-effects analysis with multiple comparisons. *$p<0.05$, ns: not significant.

(**C, D**) Shown are representative LFB stained spinal cord tissue sections (**C**) and the quantification of demyelination (LFB Score, individual data point with median+/-SEM) in the indicated treatment cohorts (n=7-8) (**D**). Statistical assessment of the data was performed with an ordinary one-way ANOVA with Dunnett`s multiple comparison test.**$p<0.01$. Scale bars in (C) represents 100µm.

Supplemental material:

Material and methods:

Sex as a biological variable

This study focused on female mice as women are more prone to develop severe forms of autoimmune diseases such as multiple sclerosis or rheumatoid arthritis modelled in this study.

Mice

KBxN mice were generated by crossing KRN transgenic mice with NOD-Shild mice as described (1). KBxN mice were bred and maintained at Charles River France. C57BL/6 mice used in the experimental autoimmune encephalomyelitis (EAE) model were provided by Shanghai BK/KY Biotechnology Co., Ltd, Shanghai, China. All mice were maintained according to the rules and regulations of the local animal facilities in France, Germany, and China.

Antibodies

IL23 (p19)-specific human IgG1, human IL23-IgG1-F241A, and human TNP-specific IgG1-F241A antibodies as well as the human IgG1-Fc-F241A antibody fragment (NVG-2089) were produced by ATUM (Newark, California, USA), Just-Evotec Biologics (Seattle Washington, USA), or Evitira (Schlieren, Switzerland). Antibodies or antibody fragments were produced in HD-BIOP3 GS Null Chinese hamster ovary (CHO) K1 cells, stably expressing the respective antibody heavy and light chains. To generate highly sialylated TNP-IgG1-F241A, IgG1-F241A-Fc and IL23-IgG1-F241A antibodies, HD-BIOP3 GS Null Chinese hamster ovary (CHO) K1 cells were co-transfected with human ST6GAL1 and human B4GALT1 to add 2,6 linked sialic acid residues (2). ATUM performed cell line construction and single cell cloning. Binding of these antibodies to mouse IL23 p19 was determined by using a sandwich ELISA. In brief, a rabbit anti-mouse p40 antibody (Clone # 2329A, R&D Systems, Minneapolis, MN) was coated to 96 well plates at 1μg/ml overnight at 4°C in 0.2M carbonate/bicarbonate buffer. Wells were

then washed and blocked with 5% BSA in PBS-0.05% tween for 1 hour at room temperature, followed by incubation with 0.25μg/ml mouse IL23 (R&D Systems, Minneapolis, MN) suspended in 1% BSA in PBS-0.05% tween for an additional 1 hour at room temperature. Plates were washed and incubated with the human IgG1 anti-mouse IL23 (p19) antibodies (bearing IgG Fc of Fc-F241A) or human IgG1 anti-TNP-F241A antibody at the concentrations indicated on the graph for 1 hour at room temperature. Plates were again washed and antibody binding to mouse IL23 was detected using a 1:100,000 dilution of anti-human IgG-Fc (goat Fab'2) HRP conjugate (Catalogue # 109-036-170, RRID # AB_2783740, Jackson ImmunoResearch Laboratories, West Grove,PA) followed by detection with TMB solution incubated 10 minutes, stopped using H2S04, and read using an optical density of 450nm.

Pharmacokinetic studies of the human antibodies in mice

Female BALB/c mice were dosed intravenously with human IL23-IgG1-F241A, IL23-IgG1, IgG1-F241A-Fc, or TNP-IgG1-F241A antibodies at 50 mg/kg. Blood samples were collected at various time points via retro-orbital bleeding. Serum concentrations of dosed antibodies were measured using an anti-human IgG Fc sandwich ELISA. 96-well plates were coated overnight at 4°C with anti-human IgG, Fc fragment specific capture antibody (Jackson ImmunoResearch Laboratories, West Grove, Pennsylvania) diluted to 1 µg/mL in 0.05 M carbonate/bicarbonate buffer. Plates were washed and blocked with 5% BSA in PBS-0.05% Tween-20 for 2 hours at room temperature. After washing, samples and standards prepared in 1% BSA in PBS-0.05% Tween-20 were incubated with plates for 2 hours at room temperature. Plates were washed and incubated with anti-human IgG Fc-fragment specific HRP conjugated antibody (Jackson ImmunoResearch Laboratories, West Grove, Pennsylvania) prepared at 1:50,000 in 1% BSA in PBS-0.05% Tween-20 for 1 hour at room temperature. Detection was performed using TMB solution incubated for 5 minutes, stopped with H_2SO_4, and optical density was measured at 450 nm.

Experimental autoimmune encephalomyelitis (EAE) model system

Weight matched female C57BL/6 mice (6-8 weeks of age) were used in all experiments. To induce EAE, mice were immunized with 200 µL of MOG peptide (amino acids 35-55) emulsion subcutaneously to induce EAE. In the beginning and 48 hours after immunization, each mouse was administrated intraperitoneally with 250 µL pertussis toxin (PTX, 1 µg/mL). Once the first signs of disease became apparent (clinical score below 1), animals were randomly distributed into the following experimental groups and injected every 5th day: vehicle group, IL23-IgG1 (25 mg/kg) group, IL23-IgG1-F241A (25 mg/kg), IL23-IgG1-F241A (10 mg/kg), and IL23-IgG1-F241A (5 mg/kg). All antibodies were administered by intravenous injection into the tail vein. During the experiment, the health status of the animals was monitored, the body weight and clinical score were recorded daily by an investigator blinded for the animal groups. The clinical scoring was performed as described. In brief the following symptoms and associated scores were recorded: Score 0: normal mice, no disease characteristics; Score 1: Tail weakness or slight hind limb weakness; Score 2: Tail weakness and hind limb weakness; Score 3: Unilateral hind lib hemiplegia, passive roll over cannot be recovered; Score 4: Complete paralysis of hind limbs, paralysis of forelimbs or weakened muscle strength, accompanied by urinary and fecal incontinence. Score 5: Animals need to be sacrificed due to animal welfare. On day 25 all animals were euthanized with CO_2 and tissue collected for final analysis.

Detection of demyelination in spine tissue of EAE mice.

Demyelination of spine tissue was revealed by luxol fast blue (LFB) staining. In brief, spine tissue was fixed in 10% formalin for 48 hours followed by removal of the backbone. Spinal cord tissue was dehydrated by ascending ethanol solutions and embedded in paraffin. 4µm tissue sections were cut with a rotary microtome and dried at 60°C for two hours before staining with 0.1% LFB solution overnight at 56°C. Areas with decreased LFB staining were identified as areas with demyelination and assigned scores (Score 0: no demyelination; Score 1: one small area of demyelination; Score 2: 2 or 3 small areas of demyelination; Score 3: 1 or 2 large areas of demyelination; Score 4: extensive demyelination larger than 20% of the white matter area) by an investigator blinded for the samples.

KBxN model system

KBxN mice develop a spontaneous rheumatoid arthritis starting around three weeks of age. All animals with clinical scores below 2 were included in the experiment and randomly assigned to groups. Arthritis was scored by clinical examination as described by an investigator blinded for the experimental groups (2). In brief, the index of all four paws was added: 0 (unaffected), 1 (swelling of one joint), 2 (swelling of more than one joint), and 3 (severe swelling of the entire paw), accumulating to a maximum score of 12. Once the first signs of RA became apparent (score below 3), animals were separated into experimental groups and treated with PBS or the respective IL23-specific IgG1 and IgG1-F241A antibodies, the IgG1-F241A Fc, or combinations of antibodies at three day intervals by intraperitoneal injection for twelve days.

Bone histomorphometry

Histological analysis was performed by an investigator blinded for the experimental groups. To quantify bone erosions, osteoclast numbers, and osteoclast size, all tissue was removed from hind legs followed by decalcification for 2 weeks in 14% (wt/vol) EDTA (pH adjusted to 7.2 by addition of ammonium hydroxide). 5μm paraffin sections of the tibia and the paw were stained for TRAP and quantification of the different parameters was done by digital image analysis (OsteoMeasure; OsteoMetrics).

Analysis of antibody glycosylation

Releasing and labelling N-glycans

N-glycan analysis for all proteins was performed by ATUM using standard methodology. N-glycans were released and labeled using the InstantPC kit (Agilent). In brief, 20 mg of intact mAB or fragments and 2ml of Gly-x denaturant was added. The samples were incubated at 90°C for 3 minutes, and then the samples were allowed to cool at room temperature for 2 minutes. 2ml of N-glycanase was added to each sample, mixed and then incubated at 50°C for 5 minutes. 5ml of InstantPC Dye solution was mixed with the samples and incubated for a

further 1 minute at 50°C. To each sample, 150ml of load/wash solution was applied. The samples were transferred to a clean-up plate and vacuum applied. The samples were washed three times with load/wash buffer before the released labeled N-glycans were eluted with 100ml of Gly-X Instant PC. The samples were either run immediately on the HPLC-HILIC-FLD or Agilent 6530-QTOF or stored with a foil plate seal at -20°C.

HILIC-HPLC Glycan Analysis

InstantPC labeled glycans were analyzed by HPLC-FLD on an Agilent 1290 HPLC system with a fluorescence detector (Agilent, USA) using an AdvanceBio Glycan mapping 300A column (1.8mm, 2.1 x 150mm, Agilent) with an increasing ammonium formate linear-gradient (mobile phase A: 100mM ammonium formate pH 4.5 in water; mobile phase B: Acetonitrile) at a flow rate of 0.6mL/min. This column allows detection of 2,3 as well as 2,6 linked sialic acid isoforms. An injection volume of 2ml and a column temperature of 40°C were used. Glycans were detected at a wavelength of 345nm with an excitation wavelength of 285nm. Peaks were integrated using OpenLabs CDS software (Agilent), and the relative glycan compositions were calculated. In conjunction with the samples being run, a dextran ladder (AdvanceBio InstantPC Maltodextrin ladder; Agilent) was run before and after the samples. A ladder was used to calibrate the LC runs and to plot a curve to allocate GU values from retention times. Calculated GU values were compared to a database of reference structures (InstantPC Labeled Glycans, Agilent), allowing N-Glycan to be assigned to each peak. The relative abundance (%Area) of each glycan is expressed as the average of the percentage of the total peak area.

Glycan Analysis via Mass Spectroscopy

Purified IgG1 N-glycopeptides were analyzed on a Waters nano ACQUITY UPLC system (Waters, Milford, MA, USA) coupled to a Bruker Compact Q-TOF mass spectrometer (Bruker Daltonics, Bremen, Germany) equipped with a Captive Spray ion source. Samples were separated on a C18 nano-LC column (150 mm × 100 μm, 2.7 μm particle size) using a linear gradient of 10–45 % mobile phase B (80 % ACN, 0.1 % TFA) over 7.5 min. Mass spectra were acquired over an m/z range of 600–2000 at a rate of 0.5 Hz. Raw data were processed using

Bruker Data Analysis 4.1, and glycopeptide assignments and relative quantification were performed using the semi-automated LacyTools software suite. Manual data curation was then performed to exclude analytes with low mass accuracy, signal-to-noise ratio and isotopic pattern quality from further analysis.

Statistical analysis

Data is shown as Mean ± SEM and was analyzed by GraphPad Prism with T-Test, One Way ANOVA or Two Way ANOVA (* $P<0.05$, ** $P<0.01$, *** $P<0.001$; ns: not significant).

Study approval

Animal experiments and animal specimen analysis were performed by BioDuro Co., Lt. in Jiangsu, China and at the FAU Erlangen-Nürnberg and were reviewed by institutional review boards in China and Germany (approved under license NIG-FFS-PH-20240809-01 and 55.2.2-2532-2-2170-21).

Data availability

All data and the “Supporting data values” file is available from the corresponding authors upon request.

Acknowledgement

This study was funded by NUVIG therapeutics and grants from the German Research Foundation (DFG-FOR2886-B2, DFG-FOR2953-P3, DFG-TRR369-C01) to FN.

Additional references:

1. Kouskoff V, Korganow AS, Duchatelle V, Degott C, Benoist C, and Mathis D. Organ-specific disease provoked by systemic autoimmunity. *Cell.* 1996;87(5):811-22.
2. Ji H, Ohmura K, Mahmood U, Lee DM, Hofhuis FM, Boackle SA, et al. Arthritis critically dependent on innate immune system players. *Immunity.* 2002;16(2):157-68.

3. Seeling M, Hillenhoff U, David JP, Schett G, Tuckermann J, Lux A, et al. Inflammatory monocytes and Fcgamma receptor IV on osteoclasts are critical for bone destruction during inflammatory arthritis in mice. *Proc Natl Acad Sci U S A.* 2013;110(26):10729-34.
